# 12 h Abstinence-Induced ERP Changes in Young Smokers: Electrophysiological Evidence From a Go/NoGo Study

**DOI:** 10.3389/fpsyg.2019.01814

**Published:** 2019-08-14

**Authors:** Chang Liu, Fang Dong, Yangding Li, Yan Ren, Dongdong Xie, Xianfu Wang, Ting Xue, Ming Zhang, Guoyin Ren, Karen M. von Deneen, Kai Yuan, Dahua Yu

**Affiliations:** ^1^Inner Mongolia Key Laboratory of Pattern Recognition and Intelligent Image Processing, School of Information Engineering, Inner Mongolia University of Science and Technology, Baotou, China; ^2^Guangxi Key Laboratory of Multi-Source Information Mining and Security, Guangxi Normal University, Guilin, China; ^3^School of Life Sciences and Technology, Xidian University, Xi’an, China

**Keywords:** event-related potentials, young smokers, 12-h abstinence, Go/NoGo task, inhibition control

## Abstract

Decreased inhibition control ability and increased craving may be the most important causes of relapsing in smoking. Although inhibition control defects in young smokers were investigated, the effects of short-term abstinence on inhibition control in young smokers were still unclear. Thirty young smokers participated in the present study. The EEG signals during the Go/NoGo task were recorded in both satiety and 12 h abstinence conditions. The task performances were observed and compared between the two conditions. Event-related potential (ERP) analysis was used to investigate changes in N200 and P300 amplitude and latency induced by 12 h of abstinence. After 12 h of abstinence, the latency of N200 was prolonged in young smokers. No significant changes were found in the number of NoGo errors and the response time of Go in young smokers after 12 h of abstinence. Correlation analysis showed that the N200 latency of abstinence condition was significantly correlated with the number of NoGo errors and the response time of Go in the abstinence condition. The present findings may improve the understanding of the effect of short-term abstinence in young smokers. We suggested that the latency of N200 may be associated with inefficient inhibitory control of the abstinence condition in young smokers. Our results may contribute new insights into the neural mechanism of nicotine abstinence in young smokers.

## Introduction

Smoking is the leading cause of death in the world, which may cause 6 million people to die annually (World Health Organization, 2013). “China Report on the Health Hazards of Smoking” in 2012 indicated that China had 350 million smokers including 14 million young smokers and more than 1 million deaths each year due to smoking-related diseases. A national survey showed that young adults had the highest smoking rate than any age group in the United States (Ling et al., 2009). In addition, at least 20% of young smokers which had higher levels of nicotine dependence became regular smokers (Lantz, 2003). Previous studies of young smokers reported that nicotine may affect brain maturation and nervous system development in adolescents (Galván et al., 2011; Li et al., 2015; Bu et al., 2016; Yuan et al., 2016a, 2017, 2018; Su et al., 2017; Wang et al., 2017; Yu et al., 2017a, b; Zhang et al., 2017). Although most young smokers are aware of the negative consequences of smoking and expressed a strong desire to quit smoking, the vast majority of quitting attempts ended in relapse (Kenford et al., 2002). Previous studies found that smokers had nicotine withdrawal symptoms during the withdrawal state as increased craving and decreased cognitive control ability, which may lead to the failure of smoking cessation and even relapse (Jacobsen et al., 2004; Lerman et al., 2014). On average, the percentage of smokers who successfully quit more than one year will not exceed 5% (Jamal et al., 2014). The changes of craving and cognitive control during the short-term abstinence state may improve our understanding of smoking for young people (Cahill et al., 2012).

Cognitive impairment is related to nicotine abstinence, which may be an important cause of relapse (Beaver et al., 2011; Yuan et al., 2016b). Previous studies in our group confirmed there were functional changes in the brain caused by 12-habstinence in young smokers (Li et al., 2016; Bi et al., 2017; Kai et al., 2018; Zhao et al., 2018). However, there are few studies on brain electrophysiological changes caused by 12-h abstinence in young smokers. As a reliable method, the utilization of a longitudinal experiment design not only increased statistical power, but also allowed for the examination of correlations between changes in inhibition control, and changes in brain activation across sessions (i.e., satiety vs. smoking abstinence) (Borst et al., 2014; Paulsen et al., 2015). In contrast to fMRI technique measuring hemodynamic response, the EEG/ERP technique captures electrophysiological signals directly related to neuronal activity. Present study mainly explored the changes of inhibitory control and electrophysiological changes induced by 12 h of abstinence in young smokers.

**FIGURE 1:**
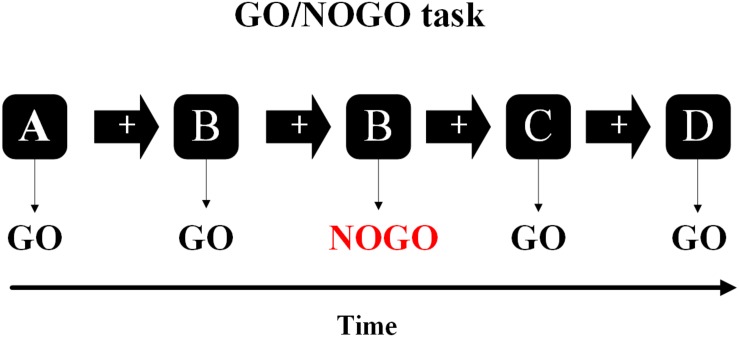
In the Go/NoGo task, the subjects needed to respond to one stimulus (Go-stimuli) and to refrain from responding to the other stimuli (NoGo-stimuli).

In the current study, the inhibitory cognitive control ability of young smokers was measured by a Go/NoGo task. In this task, participants responded quickly to “Go” stimuli without responding to “NoGo” stimuli, and the more they responded to “NoGo” stimuli, the more serious their inhibitory cognitive control deficits (Figure 1; Littel et al., 2012). Event-related potentials (ERP) were used to investigate the changes of NoGo-N200 and NoGo-P300 amplitude and latency of Go/NoGo task between satiety and 12 h abstinence sessions in young smokers. Two major ERP components, N200 and P300, have been shown to be associated with smoker inhibition control in the Go/NoGo task (Bokura et al., 2001; Luijten et al., 2011; Cid-Fernández et al., 2014). Among them, P300 is a positive wave about 300 ms after the reaction occurs, which is related to the actual inhibition process (Falkenstein et al., 1999; Cheng et al., 2016). N200 is a negative wave about 200 ms after the reaction occurs, which is related to the conflict detection during the early stage of the inhibition process (Donkers and van Boxtel, 2004). We hypothesized that the electrophysiological measurements and behavior of young smokers would change after 12 h of abstinence during the Go/NoGo task, and these changes were related to the inhibition control impairments in young smokers.

## Materials and Methods

### Participants

Thirty male smokers (19–21 years of age) were recruited from Inner Mongolia University of Science and Technology in the present study. All smokers had normal vision. The selection criteria for young male smokers who have never quit smoking before were as follows: (1) reported smoking more than 10 cigarettes a day in the last 6 months; (2) met the DSM-V criteria for current nicotine dependence; and (3) no smoking cessation in the past year. Additionally, nicotine dependence levels were assessed with the Fagerstrom Test of nicotine dependence (FTND) (Fagerstrom and Schneider, 1989). (4) Participants were right-handed as measured by the Edinburgh Handedness Inventory (Oldfield, 1971). (5) Had no physical illness or neurological/psychiatric disorders as assessed by clinical evaluations and medical records, or alcohol/drug abuse. All participants were already familiar with the experimental process and signed the written informed consent. More detailed information can be found in Table 1.

**TABLE 1:** Demographic characteristics of young smokers in the present study.

**Clinical details**	**Young smokers (*n* = 30)**
Age (years)	20.9 ± 1.19
Age range (years)	19 – 21
Education (years)	14.06 ± 0.57
Age of smoking initiation	15.2 ± 2.76
Smoking duration (years)	4.31 ± 2.19
Pack-years	3.13 ± 2.49
Cigarettes per day (CPD)	14.27 ± 4.35
FTND total score	4.73 ± 1.59

*Values are expressed as means ± standard deviations. FTND, Fagerström Test for nicotine dependence; Pack-years, smoking years × daily consumption/20.*

### Procedures

Participants filled out questionnaires on demographics. We used a within-subject design with two identical EEG sessions occurring 1–3 weeks apart: (1) smoking as usual (satiety) and (2) ≥12 h abstinence (abstinence). The smoking satiety participants could smoke within 20 min before the experiment. During abstinence, the participants were banned from using cigarettes, alcohol or other drugs (including caffeine and nicotine) at least 12 h prior to the experiment. Before starting the experiment, the participants sat comfortably and the electrodes were connected. Participants were told that mistakes are inevitable, but they should do their best to complete the task as quickly and accurately as possible. Then, all participants were asked to complete the Go/NoGo task and their EEG data were collected simultaneously. This study used E-prime 2.0 software (Psychology Tools, Pittsburgh, PA, United States) to collect participants’ behavior data. The authenticity of the 12-h abstinence was confirmed by the expiratory carbon monoxide (CO) levels measured using the Smokerlyzer system (Bedfont Scientific Ltd., Rochester, United Kingdom). Specific measurement criteria were that all subjects had to have their CO levels measured 10 min before the beginning of the experiment. The participants were required to stand up and hold the Smokerlyzer system in their right hand. When the Smokerlyzer system began to detect CO, the participants were asked to breathe out CO into the Smokerlyzer system with their mouth. CO level in the expired air was verified as ≤8 parts per million (ppm) during the abstinence state, which showed a distinct reduction for each participant compared to that measured during the satiety state (>10 ppm) (Table 2; Heimstra et al., 1980).

**TABLE 2:** The age of each smoker and the CO in both sessions (satiety and abstinence).

**Subject ID**	**Items**		
	**Age (years)**	**CO (ppm)**	
		**Satiety**	**Abstinence**
1	20	11	7
2	20	13	4
3	20	10	6
4	21	11	2
5	21	10	3
6	20	12	5
7	20	18	4
8	19	12	3
9	21	14	5
10	20	11	3
11	21	12	2
12	21	14	2
13	20	13	5
14	20	16	6
15	20	13	5
16	19	15	3
17	21	12	4
18	21	14	2
19	19	11	5
20	20	10	3
21	21	12	8
22	21	15	7
23	21	12	2
24	20	12	2
25	20	19	7
26	21	12	2
27	20	13	4
28	20	15	4
29	20	10	4
30	21	11	4

### Task Paradigm

The Go/NoGo task was divided into four sections, each consisting of 159 letters (e.g., A, B, C, and D), with 60 s of rest after each section. About 74 stimulations (NoGo trials) were randomly inserted into the task and the stimulation was never continuous. The letter appears in the middle of the screen for 600 ms, preceded by a white cross against a black ground for 300 ms. Participants were asked to respond with their right index finger quickly to the stimulus (Go trials), but remained silent on the repeated stimulation (NoGo trials) (Yin et al., 2016).

### EEG Recording and Data Analysis

The electroencephalogram (EEG) recordings were performed in a closed, quiet room. The participants were arranged to be 100 cm away from the screen to ensure that the display was clearly visible. EEG data were recorded by using the BrainAmp MR plus (Brain Products GmbH. Munich. Germany) with the electrodes at 64 scalp sites (positioned following the 10–20 International System) with an additional electrode at FCz (reference electrode). The vertical electrooculogram (EOG) of the two electrodes was recorded at a position above the left eye and at an outer corner of the right eye. All signals were digitalized with a sample rate of 1000 Hz with a frequency band from 0.10 to 250 Hz, and impedances were reduced to less than 10 kΩ. All offline data were processed using Brain Vision Analyzer 2 (Brain Products GmbH. Munich. Germany). The EEG data re-referenced the mean values of the mastoids (TP9 and TP10). EEG signals were band-pass filtered using a 0.15–30 Hz (IIR filter 24 dB/octave roll off, 50 Hz notch) band-pass filter. Eye movements and eye blinks were removed using an independent component analysis (ICA). Raw data inspection was done for all epochs (−200 ms pre-stimulus to 800 ms post-stimulus). The rejection criteria were: maximal allowed voltage step (gradient) was 50 μv for each sample point, maximal allowed amplitude was ∼75∼75 μv, maximal allowed value difference was 100 μv in a 200 ms interval and activity below 0.5 μv in a 100 ms interval was rejected. All data were then baseline corrected from −200 to 0 ms pre-stimulus. Average of the data for a specific epoch was done according to the condition (NoGo). Based on previous studies, the N200 and P300 components were analyzed at PZ and FCz electrode points (Tian and Yao, 2008). The N200 and P300 amplitudes were defined as the global maximal value to baseline at the signal subject level (N200, 150∼300 ms post-stimulus; P300, 250∼400 ms post-stimulus) (Littel et al., 2012).

### Statistics

Statistical analysis of all data was performed using the Statistical Product and Service Solution 20 (SPSS 20). Two-sample paired *t*-test was used to detect differences in behavioral data (NoGo errors, Go reaction time) and ERP data (amplitude and latency of the N200 and P300) between the satiety session and abstinence session. Pearson correlation coefficients were calculated for behavioral data (NoGo errors, Go reaction time), smoking status [pack-years, FTND, cigarettes per day (CPD)] and both latency, and amplitude of ERPs (N200 and P300).

## Results

### EEG Data

#### N200

**FIGURE 2:**
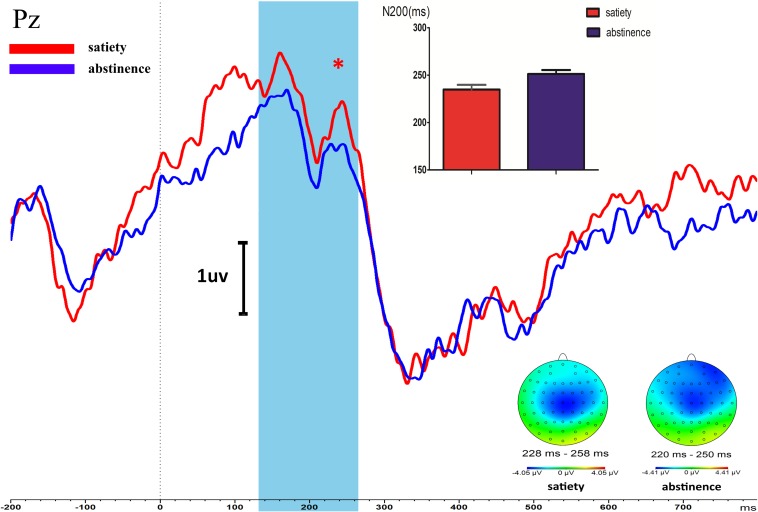
Longer NoGo N200 latency of PZ was found in young smokers after 12-h abstinence (12-h abstinence: 234.96 ± 26.84, satiety: 251.33 ± 22.62; *p* = 0.018). The NoGo P300 amplitudes and latency were not different between the 12-h abstinence smoking condition and smoking satiety condition. ^*^Represents an area where there are significant differences in ERP between satiety and abstinence in young smokers.

After 12 h of abstinence, the latency of N200 was prolonged compared with that in the satiety sessions during the Go/NoGo task state in young smokers (*t* = −2.517, *p* = 0.018). The amplitude of N200 in 12-h smoking abstinence condition had an increasing trend compared with the smoking satiety sessions in young smokers but the difference was not significant (*t* = −0.904, *p* = 0.37) (Figure 2).

#### P300

There was no significant difference between the smoking satiety group and the 12-h smoking abstinence session on amplitude (*t* = −0.851, *p* = 0.402) and latency (*t* = 0.307, *p* = 0.716) of P300 (Figure 2).

### Behavioral Data

**FIGURE 3:**
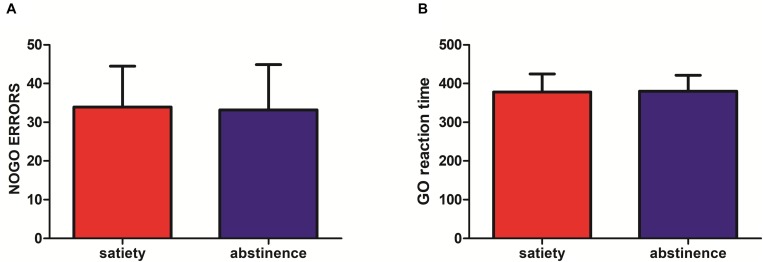
**(A)** No significant difference was found in NoGo errors (12-h abstinence: 33.20 ± 11.70, satiety: 33.93 ± 10.55; *p* = 0.8) after 12-h abstinence. **(B)** No significant difference was found in Go reaction time (RT) (12-h abstinence: 380.16 ± 41.17, satiety: 377.96 ± 46.84; *p* = 0.84) after 12-h abstinence.

No significant difference was found in the Go response time (RT) (12-habstinence: 380.16 ± 41.17, satiety: 377.96 ± 46.84, *p* = 0.84) and NoGo errors (12-h abstinence: 33.20 ± 11.70, satiety: 33.93 ± 10.55; *p* = 0.8) between the smoking satiety session and the 12-h smoking abstinence session (Figure 3).

#### Correlations

**FIGURE 4:**
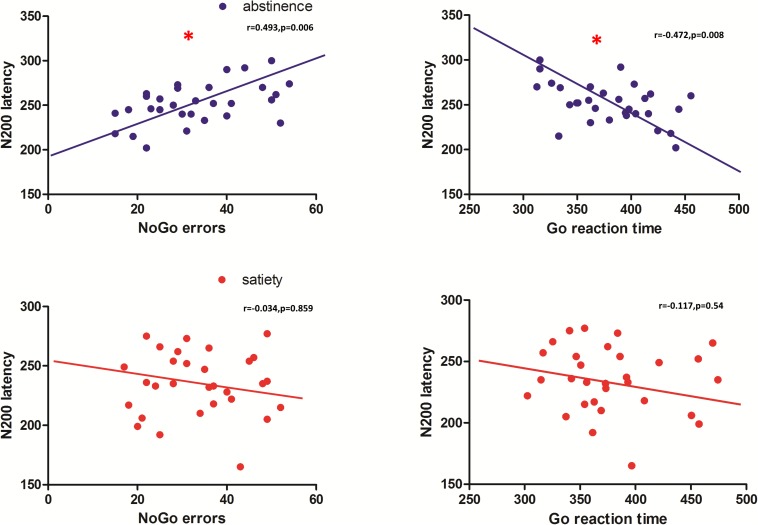
N200 latency of the abstinence condition was significantly correlated with the number of NoGo errors (*r* = 0.493, *p* = 0.006) and the response time of Go errors (*r* = 0.472, *p* = 0.008). No significant correlation was found in the smoking satiety condition. ^*^Represents that there is a significant correlation between NoGo errors, the response time of Go errors and the N200 latency.

Pearson correlation coefficients were calculated for the behavioral data and smoking status (pack-years, FTND, and CPD) and both latency and amplitude of the NoGoN200 and P300. For the 12-h smoking abstinence session, a significant correlation was found between NoGo errors and N200 latency (r = −0.487, *p* = 0.006), and a significant correlation was also found between the Go response time and N200 latency (*r* = −0.472, *p* = 0.008) (Figure 4). No significant correlation was found between the electrophysiological measurements and clinical measures, pack-years, cigarettes per day, and FTND. Furthermore, a correlation analysis replicated previous results showing a significant correlation between the P300 amplitude and NoGo errors during the smoking satiety session (*r* = 0.370, *p* = 0.044).

## Discussion

Young smokers may become lifelong smokers which will make it difficult to quit smoking in the future. The failure of young smokers to quit smoking is probably related to inhibition control (Lubman et al., 2015). The weakening of the inhibition control ability may make them unable to control their desire to smoke leading to relapse. Studying the critical moment of early smoking abstinence in adolescent smokers, especially 12 h of smoking abstinence, is likely to help us understand the neurological mechanism of smoking. For example, the latest study in our group found that the tract strength of the left striatum-dorsolateral PFC (DLPFC) during the 12-h abstinence predicted the lapse in smokers with an accuracy of 68.3% (Kai et al., 2018). Event-related potential (ERP) can accurately detect changes in brain-evoked potential under different events due to its high temporal resolution, providing the flow of information almost in real time (Falkenstein et al., 1999; Gajewski and Falkenstein, 2013; Kamarajan and Porjesz, 2015). Therefore, we employed the ERP method to compare inhibition control ability between 12-h abstinence vs. satiety conditions in young smokers whose ERP components are usually quantified by their amplitude and latency measures. N200 and P300 each reflect unique cognitive brain functions (e.g., attention, motivation, and higher level executive function) (Patel and Azzam, 2005; Parvaz et al., 2011; Gajewski and Falkenstein, 2013). In Go/NoGo tasks, the more NoGo errors, the weaker the inhibition and control ability, while the longer the response time of Go, and the worse the response performance to the task (Luijten et al., 2011; Littel et al., 2012). In the present study, we found that the latency of N200 was prolonged in young smokers after 12 h of abstinence. In addition, previous research by our group found that young smokers were deficient in the inhibition control ability and the P300 amplitude of young smokers was lower than that of non-smokers (Yin et al., 2016). However, the 12-h abstinence did not make the young smokers’ inhibition control deficits more serious. In contrast, another study found that short-term abstinence could lead to reduced heart rate, worse task performance, feelings of depression, stress, irritability, restlessness, poor concentration, and urges to smoke (Parrott et al., 2015). Moreover, the correlation analysis showed that there was a significant correlation between N200 latency and NoGo errors and Go response time after 12 h of abstinence in young smokers.

It was demonstrated that N200 represented the process of reaction inhibition or conflict monitoring (Donkers and van Boxtel, 2004; Bekker et al., 2005; Kaiser et al., 2006). In the Go/NoGo task, the NoGo-N200 was related to increased effort in activating the response inhibition system that interrupted preparations for response execution (Géczy et al., 1999; Bokura et al., 2001; Tian and Yao, 2008). In addition, related research showed that ERP indices have been used to predict relapse (Parvaz et al., 2011). For example, ERP studies in sober alcoholics found delayed N200 latency to distinguish between abstainers and relapsers with an overall predictive rate of 71% (Glenn et al., 1993). Furthermore, it has been suggested that N200 latency is an indicator of the rate of inhibition of control response preparation, and is related to cognitive monitoring efficiency (Falkenstein et al., 1999; Gajewski and Falkenstein, 2013). For instance, in a study observing behavioral responses of activation and inhibition processes in children, adolescents, and adult populations, the better the task performance, and the longer the latency of N200 (Johnstone et al., 2005). In another study comparing adolescent smokers with non-smokers, smokers exhibited a reduced N200 component with no behavioral deficits suggesting that N200 may provide a sensitive index of cognitive control deficits in smokers (Buzzell et al., 2014). Therefore, the abnormality of the N200 component, especially the abnormality of the N200 latency, is likely to be regarded as a marker for neural deficits in inhibition control in the abstinence condition. Previous studies in our group suggested that smoking abstinence dramatically alters neural mechanisms underlying interactions, thus affecting the balance between reward and cognitive control (Li et al., 2016; Bi et al., 2017; Kai et al., 2018; Zhao et al., 2018). In the present study, the result that reduced inhibitory control ability was found in NoGo accuracy as well the N200 component changes of the ERP provides support for the hypothesis that there is a general shortcoming in response inhibition control in young smokers after 12 h of abstinence. Furthermore, N200 latency was related with NoGo errors during the abstinence condition suggesting that N200 latency may be related to the inhibition control ability in young smokers after 12 h of abstinence. The correlation between N200 latency and Go reaction time also reflects that N200 latency may be related to reaction performance. However, the current results do not fully support this hypothesis. It may be that reduced inhibitory control ability is the result of prolonged nicotine dependence in young smokers; for example, this may occur via abnormalities in the dopamine system or due to low levels of nicotine dependence (Luijten et al., 2011). In the future, we will further explore the relationship between N200 and inhibition control ability in young smokers after abstinence for 12 h.

P300 is closely related to the actual inhibition process of the reaction (Evans et al., 2010; Littel et al., 2012; Gajewski and Falkenstein, 2013; Cid-Fernández et al., 2014). Consistent with our previous studies, the present study found that the P300 amplitude of young smokers had a significant correlation with NoGo errors during the satiety state (Evans et al., 2010; Yin et al., 2016). This finding again demonstrates the relationship between P300 and the inhibition control ability of young smokers. Unfortunately, in our study, the P300 amplitudes and latency of smoker did not significantly change after 12 h abstinence. This may be caused by a much shorter abstinence time and insufficient sample size. In the future, longer abstinence time and insufficient number of samples can be applied to further address this issue.

### Limitations

First, only young male smokers participated in the experiment and the sample size was in sufficient which may not be a representative sample of young smokers. Second, our experiments did not show changes in the control inhibition ability of young smokers after 12 h abstinence, which may be due to a much shorter abstinence time. In the future, more samples will be used for future research and female smokers will be added. In addition, a longer abstinence time experiment design will be used for the next study.

## Conclusion

In the present study, we found ERP changes in young smokers after 12-h abstinence. The longer N200 latency and the NoGo N200 latency were correlated with NoGo errors and Go reaction time during the Go/NoGo task. The present findings improved the understanding of the effect of acute smoking abstinence on electrophysiology which may contribute new insights into the neural mechanism of nicotine abstinence.

## Data Availability

All datasets generated for this study are included in the manuscript and/or the supplementary files.

## Ethics Statement

The procedures of this study were approved by the Medical Ethical Committee of the First Affiliated Hospital of Baotou Medical College, Inner Mongolia University of Science and Technology, and were conducted in accordance with the Declaration of Helsinki. All participants and their legal guardians signed in an informed consent after understanding the purpose of our research.

## Author Contributions

DY, KY, and YL conceived and designed the experiments. DX, XW, TX, MZ, and GR performed the experiments. CL, FD, DX, and XW analyzed the data. CL and FD wrote the manuscript. TX, YR, DY, KY, and KD provided the critical revision of the manuscript. All authors critically reviewed the content and approved the final version for publication.

## Conflict of Interest Statement

The authors declare that the research was conducted in the absence of any commercial or financial relationships that could be construed as a potential conflict of interest.
